# Young and middle‐aged mouse breathing behavior during the light and dark cycles

**DOI:** 10.14814/phy2.14060

**Published:** 2019-04-19

**Authors:** Candace N. Receno, Brianna E. Eassa, Caitlin M. Cunningham, Lara R. DeRuisseau

**Affiliations:** ^1^ Department of Biological Sciences Le Moyne College Syracuse New York; ^2^ Department of Mathematics, Statistics and Computer Science Le Moyne College Syracuse New York

**Keywords:** Hypercapnia, hypoxia, hypoxic hypercapnia

## Abstract

Unrestrained barometric plethysmography is a common method used for characterizing breathing patterns in small animals. One source of variation between unrestrained barometric plethysmography studies is the segment of baseline. Baseline may be analyzed as a predetermined time‐point, or using tailored segments when each animal is visually calm. We compared a quiet, minimally active (no sniffing/grooming) breathing segment to a predetermined time‐point at 1 h for baseline measurements in young and middle‐aged mice during the dark and light cycles. Additionally, we evaluated the magnitude of change for gas challenges based on these two baseline segments. C57BL/6JEiJ x C3Sn.BliA‐*Pde6b* ^*+*^/DnJ male mice underwent unrestrained barometric plethysmography with the following baselines used to determine breathing frequency, tidal volume (VT) and minute ventilation (VE): (1) 30‐sec of quiet breathing and (2) a 10‐min period from 50 to 60 min. Animals were also exposed to 10 min of hypoxic (10% O_2_, balanced N_2_), hypercapnic (5% CO_2_, balanced air) and hypoxic hypercapnic (10% O_2_, 5% CO_2_, balanced N_2_) gas. Both frequency and VE were higher during the predetermined 10‐min baseline versus the 30‐sec baseline, while VT was lower (*P* < 0.05). However, VE/V_O__2_ was similar between the baseline time segments (*P* > 0.05) in an analysis of one cohort. During baseline, dark cycle testing had increased VT values versus those in the light (*P* < 0.05). For gas challenges, both frequency and VE showed higher percent change from the 30‐sec baseline compared to the predetermined 10‐min baseline (*P* < 0.05), while VT showed a greater change from the 10‐min baseline (*P* < 0.05). Dark cycle hypoxic exposure resulted in larger percent change in breathing frequency versus the light cycle (*P* < 0.05). Overall, light and dark cycle pattern of breathing differences emerged along with differences between the 30‐sec behavior observational method versus a predetermined time segment for baseline.

## Introduction

Barometric plethysmography is an established methodology used to quantify breathing patterns in mice (Lundblad et al. 2002; Dauger et al. 2003; Receno et al. 2018a). It is noninvasive, utilized on unrestrained mice, and may be repeated. Investigators often use barometric plethysmography to measure breathing patterns with air along with other gas mixes that challenge the respiratory system (Renolleau et al. 2001; Malik et al. 2005; Fechtner et al. 2015; Receno et al. 2018a).

**Figure 1:**
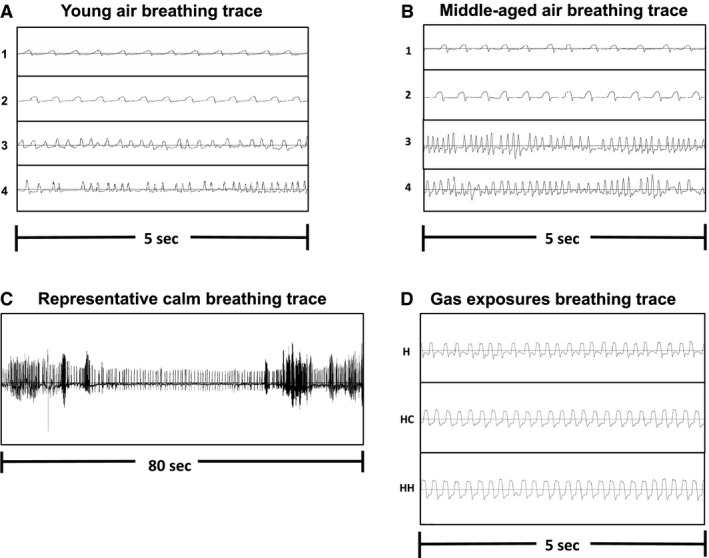
Representative breathing traces for a: (A) young mouse and (B) middle‐aged mouse during each segment of air breathing where 1 = 30‐sec light cycle, 2 = 30‐sec dark cycle, 3 = 10‐min light cycle, and 4 = 10‐min dark cycle. The *x*‐axis represents 0–5 sec and the *y*‐axis is −2 mL/min to 2 mL/min. Tracing in (C) depicts the type of data used for quiet breathing analysis, where segments of quiet breathing are flanked by increased activity. The *x*‐axis represents zero to 80 sec and *y*‐axis is −2 mL/min to 2 mL/min. (D) Representative tracings from a single mouse (middle‐aged, dark cycle) during hypoxia (H), hypercapnia (HC) and hypoxic hypercapnia (HH). The *x*‐axis represents 5 sec and the *y*‐axis is −2 mL/min to 2 mL/min.

Comparing alterations in breathing patterns during exposure to hypoxic or hypercapnic gas can be used to uncover respiratory deficiencies and/or to monitor chemosensitivity (Schlaefke et al. 1979; Gozal et al. 1994). The degree to which breathing patterns change from rest can reveal blunted, potentiated or typical responses to gas challenges. Differences in breathing may be masked depending on the baseline acquired, highlighting the importance of implementing a methodology that uses the most appropriate breathing segment with the least amount of (possible) experimental bias. One approach to defining baseline is the use of a predetermined time segment, in which mice are placed in the chamber for a period of time (e.g., 1 h) before data collection begins (e.g., the following 10–30 min) (DeRuisseau et al. 2009; Morgan et al. 2014; Perim et al. 2015). As an alternative, our group and others have made use of behavior observations for acquiring calm breathing segments, where mice are visibly awake in the chamber, but are not participating in active behaviors such as sniffing, grooming or exploring (Hickner et al. 2014; Receno et al. 2018a,b). In this case, the calm breathing segment used to evaluate the response to gas exposures (hypoxia, hypercapnia, hypoxic hypercapnia) is intended to result in a point by point comparison without the confounds of additional chamber activity. This direct comparison is possible due to the steady nature of the breaths in both segments (air, hypoxia, etc.) and similar observed behaviors (Fig. 1). For instance, during gas exposures mice typically sit quietly in the chamber. We were interested in ways to compare air breathing to hypoxia, hypercapnia and hypoxic hypercapnia that most represented a direct comparison of breathing across exposures.

Obtaining a calm baseline has its issues, as each mouse varies in how they respond to the chamber environment. This method may result in discrepancies in the amount of time it takes to obtain baseline. Some animals require more time to reach a calm state versus others, and external stimuli can play a large role in observed behaviors (Kabir et al. 2010; Teske et al. 2014). Another factor to consider is the period of the circadian cycle when mice are examined. Testing in the dark (active) phase may require more time for familiarization to the chamber compared to the light (resting) phase. Finally, the expected duration of quiet breathing can impact the feasibility of acquiring baseline measures, as it may be more challenging to successfully measure longer (minutes to hours) versus shorter (seconds) segments of calm breathing.

The overall goal of this study was to compare values obtained from different segments of air breathing across two points (light, dark) in the circadian cycle and two ages of mice. After initiating studies in middle‐aged mice in the dark cycle, we observed challenges that had not previously been revealed. Therefore, an additional rationale for this work was to standardize how mice across the lifespan and circadian cycle can be compared with limited bias. Although we have typically reported a 2–10 min baseline period (Loeven et al. 2018; Receno et al. 2018a,b) for air breathing, this time span of calm breathing was not observed in these groups of mice, even after multiple chamber habituation days and over 3 h in the chamber during testing. Based on this behavior, we aimed to find a more appropriate way to quantify air breathing that could be used to compare mice in various conditions (e.g., young, old, light, dark). While one option was to manually accept only “resting breaths” (remove sniffs/grooming/locomotor activity) during an arbitrary time‐point, this method did not seem ideal, particularly when investigating mice in the dark cycle. Using the manual method of accepting breaths was incredibly time consuming and fewer breaths were accepted in older mice during the dark cycle, so the comparison across groups was not a direct point by point comparison. With the increased use of unrestrained barometric plethysmography systems, including some programs that do not visually identify which breaths are accepted in the analysis and which are excluded, the latter method would not allow for assessments across laboratories. Therefore, we aimed to offer two methods of baseline that could be repeated. The analyses are not meant to be exhaustive, but are intended to start a broader discussion of additional ways to quantify breathing across the lifespan and circadian cycle in mice.

We tested both young and middle‐aged mice using two different criteria for baseline: a 30‐sec segment of calm, quiet breathing versus a 10‐min average at the end of the first hour in the chamber. Using these baseline criteria, we compared the magnitude of response to gas exposures in both the light and dark cycles. In a subset of mice, we also analyzed VE/VO_2_ for the time segments during baseline and hypoxic exposure. We hypothesized that analyzing 30‐sec calm breathing segments would result in lower VE at baseline compared to a predetermined time‐point. Additionally, we hypothesized that a quiet breathing baseline would reveal larger percent changes in breathing patterns during gas exposures when compared to a predefined 10‐min baseline segment at 1 h. This rationale is based on the similar behaviors (sitting, still in the chamber) observed during gas exposures and a calm baseline.

## Materials and Methods

### Animals

Mice used in this study were a subset from a larger study related to breathing patterns in wild‐type and Ts65Dn animals; only WT animals (C57BL/6JEiJ x C3Sn.BLiA‐*Pde6b* ^*+*^/DnJ; F1, stock #: 3647) were used for these experiments. However, all mice were housed in the same room. Two cohorts of male mice 3–5 months old (young, *n* = 7) and 12–15 months old (middle‐aged, *n* = 11) were utilized. All mice were housed in standard laboratory conditions (12‐h light/dark cycle) and provided food and water ad libitum.

### Ethical approval

All procedures were approved by the Le Moyne College Institutional Animal Care and Use Committee and were carried out in accordance to the policies described in the Guide for the Care and Use of Laboratory Animals (National Research Council (US) Committee for the Update of the Guide for the Care and Use of Laboratory Animals, 2011).

### Unrestrained barometric plethysmography

Whole body unrestrained barometric plethysmography was utilized as previously described (Receno et al. 2018a,b); mice were tested during hours 4–7 of the light cycle and 4–7 of the dark cycle. Briefly, mice were moved to the testing room shortly after calibration of the unrestrained barometric plethysmography set up. Light cycle testing occurred under typical laboratory lighting during hours 4–7 of the light cycle, whereas dark cycle testing was completed with all lights turned off in the room during hours 4–7 of the dark cycle. Both testing conditions had lighting similar to where animals were housed. The behavioral testing room was across the hall from the housing room, so light exposure (during the dark cycle) and transport stress was minimized. Experimenters were blinded to the animal group during plethysmography data collection and breathing trace analysis. Mice were identified via eight digit LifeChip (Destron Fearing, Airport, TX) number.

### Baseline segments

Mice were allowed to move freely within the chamber throughout the entire testing period. Initially, breathing patterns were recorded at minutes 50–60 for a 10‐min average, similar to protocols utilizing a standard habituation time; no breaths were manually excluded from flow tracing analysis. Following this time, mice were kept in the chamber until quiet breathing was documented, which was defined as a period when mice were awake with no instances of sniffing and grooming. It should be noted that our experience is that mice that are awake do not sit and rest in the chamber for long periods of time as rats do. Mice continue to explore the environment, except for brief periods which we qualified as calm breathing. This behavior is noted in Figure 1 which shows a typical response in mice; calm breathing segments were always flanked by increased respiratory activity on each side. The following segments were used for analysis: (1) 10‐min baseline (minutes 50–60) and (2) 30‐sec baseline (during quiet breathing).

### Gas exposures

Once quiet breathing baselines were established, all mice were exposed to the following conditions with each exposure lasting 10 min: hypoxic gas (10% O_2_; balanced N_2_), hypercapnic gas (5% CO_2_; 20.93% O_2_; balanced N_2_), and hypoxic hypercapnic gas (10% O_2_; 5% CO_2_; balanced N_2_). Each condition was followed by a 10‐min recovery period (20.93% O_2_; balanced N_2_). Body temperature was measured throughout the experiment via the implantable LifeChip system. If body temperature changed by ≥1°C, the temperature was adjusted in Ponemah.

### Unrestrained barometric plethysmography analysis

Analysis of breathing frequency, tidal volume (VT), and minute ventilation (VE) were performed with DSI Ponemah software using the Drorbaugh and Fenn equation (Drorbaugh and Fenn 1955). Baseline data were analyzed for: a 10‐minute average from minutes 50–60 and a 30‐sec quiet breathing average. The software was set to the following filters: minimum flow of 0.3 mL/sec, volume match of 50% and percent relaxation of 60%. The percent change from baseline to the 10‐min average during each gas exposure (hypoxia, hypercapnia, and hypoxic hypercapnia) was calculated and analyzed. The entire 10‐min gas exposure was used to encompass the complete hypoxic response.

### Metabolic measures

Metabolic data were analyzed in a subset of mice (middle‐aged, dark cycle), with STPD flows in and out of the chamber recorded (TSI Inc., Shoreview, MN). A sample from the plethysmography chamber was pulled through Nafion tubing (PermaPure LLC, Lakewood, NJ) incased in drierite (PermaPure LLC, Lakewood, NJ). During the experiment, a sample was directed into a SA‐3 analyzer (AEI Technologies, Pittsburgh, PA) to quantify F_o_O_2_. F_i_O_2_ for baseline was quantified at the start of the experiment during a 10‐min period with air flowing through an empty chamber. F_i_O_2_ for hypoxia was 10% O_2_ and confirmed during calibrations. F_i_O_2_ and F_o_O_2_ were used to calculate VO_2_ within the Ponemah software. For hypoxia, minutes 4–10 were used for the VO_2_ analysis to account for the chamber transition period between 20.93% O_2_ and 10% O_2_. This timeframe assured a 10% O_2_ composition within the chamber, as we have previously shown a maximum time of 2 min and 25 sec for the chamber to equilibrate to the new gas exposure (Loeven et al. 2018). VE/VO_2_ was analyzed for the middle‐aged dark cycle cohort, during baseline (30‐sec, 10‐min) and exposure to hypoxia, as a proof of concept study.

### Statistical analysis

Mixed model analysis of variance (ANOVA) was implemented to test for differences in baseline measures (30‐sec vs. 10‐min), where age served as a between subject factor and light cycle as a within subject factor. Body weight was included as a covariate for VT and VE analyses (Packard and Boardman 1999). Differences in percent change from baseline to each gas were also analyzed using mixed model ANOVA, where age served as between subject factor and light cycle served as the within subject factors. VE/VO_2_ during baseline and hypoxia for middle‐aged dark trials were also analyzed using a mixed model ANOVA. Paired *t*‐tests were utilized to compare frequency, VT and VE for the time segments also analyzed for VE/VO_2_ analysis. Paired samples *t*‐tests were also used to determine if temperature differed during light and dark cycle testing in the plethysmography chamber and in the home cage. *F*‐tests were employed to determine the variability between 30‐sec and 10‐min baselines during both light and dark cycles for each individual mouse. Mice were excluded from analysis as outliers if their baseline data were >3 SDs from the mean. Significance was set *a priori* at *P* < 0.05. Data are presented as mean ± SD.

## Results

### Unrestrained barometric plethysmography at baseline – 30 sec versus 10 min

**Figure 2:**
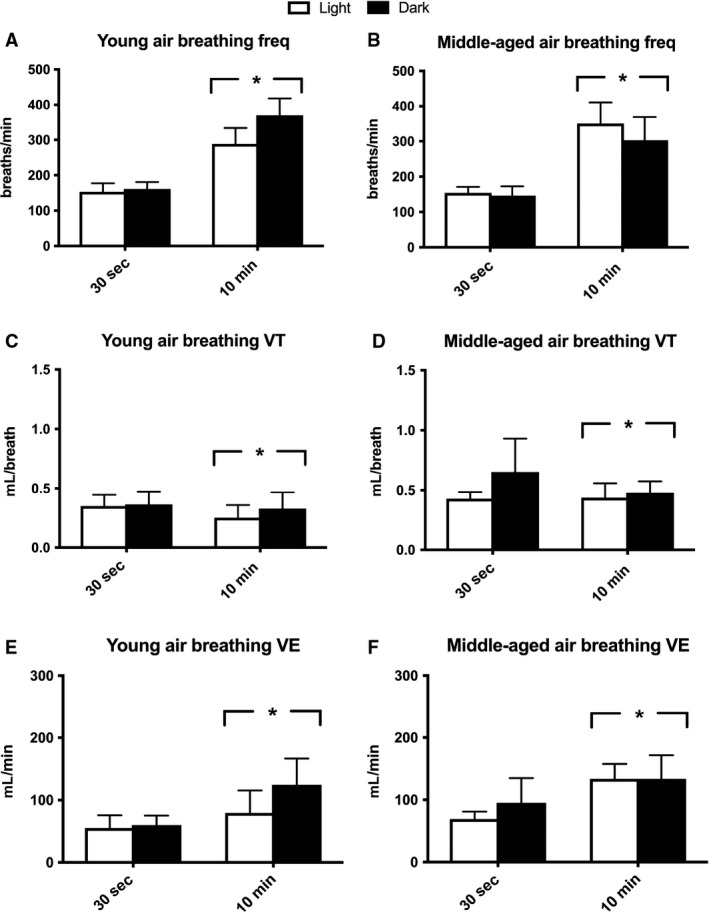
Breathing frequency (freq; breaths/min), tidal volume (VT; mL/breath), and minute ventilation (VE; mL/min) during air exposure (20.93% O_2_, balanced N_2_) in young and middle‐aged mice during a 30‐sec quiet breathing segment versus a 10‐min average analyzed from 50 to 60 min of air breathing. Quiet breathing was averaged for a 30‐sec period when mice were sitting with no locomotor movement in the cage; this often took 1–3 h to observe. Both time points were acquired during light and dark circadian cycles. Light testing period: hours 4–7 of light cycle. Dark testing period: hours 4–7 of dark cycle. Air breathing is shown for: (A) young mice (~4 months; *n* ****=** **6) frequency, (B) middle‐aged mice (~13 months; *n* ****=** **10) frequency, (C) young mice VE, (D) middle‐aged mice VE, (E) young mice VT, and (F) middle‐aged mice VT. *Significant differences were observed between 30 sec and 10 min of air breathing (*P* < 0.05) using a one‐way ANOVA. Significant differences between light and dark cycle were also detected for VT (*P* = 0.0015). Body weight was a significant covariate for both VE (*P* = 0.038) and VT (*P* < 0.001). Mice were excluded if values were > 3SD from the mean (one mouse excluded in each age group). All data are presented as mean ± SD.

Baseline frequency was significantly higher during the 10‐min baseline compared to the 30‐sec quiet baseline (*P* < 0.001; Fig. 2A and B) with no differences resulting from age (*P* = 0.734) or circadian cycle segment (*P* = 0.361). For VE measures, there was a main effect for the baseline segment used, where the 10‐min baseline showed higher VE compared to the 30‐sec calm breathing segment (*P* < 0.001; Fig. 2E and F). Body weight had a significant covariate effect among age groups for VE (*P* = 0.038). Analysis revealed that middle‐aged mice had higher VE than their younger counter parts (*P* = 0.046), which was driven by the larger body weight in the middle‐aged group. When observing VT, there was a main effect for baseline segment used, but higher values were detected for the 30‐sec baseline in this case (*P* = 0.017; Fig. 2C and D). The higher VT is likely a response to lower breathing frequencies during this 30‐sec segment. Similar to VE, body weight was a significant covariate for VT (*P* < 0.001), with middle‐aged animals having greater values. Circadian cycle also resulted in significant differences in VT, where dark cycle testing had increased values versus those in the light (*P* = 0.012). Individual *F*‐tests showed that a majority of the mice had a significantly larger variance in frequency, VT and/or VE when using the 10‐min baseline versus the 30‐sec baseline (Table 1). These data demonstrate that a calm air breathing segment results in a lower breathing frequency and minute ventilation when comparing to a 10‐min average at the end of the first hour in the chamber. Moreover, the calm segment provided an overall less variable measure of breathing pattern within each mouse. Body temperature was similar between light and dark trials (36.7 ± 0.6 vs. 36.9 ± 0.7, respectively; *P* = 0.189).

**Table 1:** Significant *F*‐tests for each mouse during 30‐sec and 10‐min baseline segments along with standard deviations for each cohort

	Light			Dark		
	Significant *F*‐tests	30‐sec SD	10‐min SD	Significant *F*‐tests	30‐sec SD	10‐min SD
Frequency						
Young	6/6	7	84	5/6	16	78
Middle‐aged	7/10	19	79	7/10	14	69
Tidal volume						
Young	6/6	0.02	0.06	4/6	0.02	0.05
Middle‐aged	6/10	0.04	0.09	8/10	0.05	0.16
Minute ventilation						
Young	6/6	3.2	19.5	6/6	5.1	21.9
Middle‐aged	9/10	6.2	28.6	8/10	6.9	30.6

Number of mice that showed increased variability in frequency, tidal volume and minute ventilation during the ten‐minute (10‐min) baseline segment versus the 30‐second (30‐sec) segment. *F*‐tests compared standard deviations of each variable in individual young (*n* ****=** **6) and middle‐aged (*n* ****=** **10) mice during both the light and dark cycle. Most mice showed increased variability of measures during the 10‐min compared to the 30‐sec segment (*P* < 0.05). Value listed for SD is the average SD for mice within the respective time segment.

### Unrestrained barometric plethysmography – gas exposures

**Figure 3:**
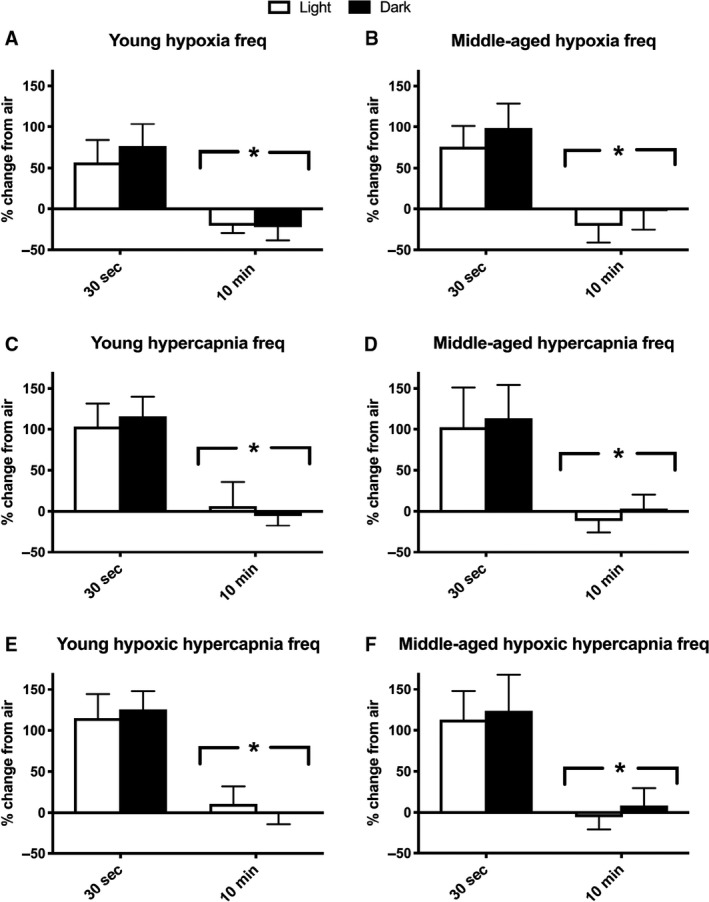
Percent change from air breathing frequency to various gas exposures is larger with use of a 30‐sec baseline. Air breathing was calculated as the 30‐sec of quiet breathing or the 10‐min average at the end of the first hour of air breathing. Each mouse's individual percent (%) change for a given gas exposure (10‐min average) was calculated. Both cohorts were tested in light and dark conditions. Light testing period: hours 4–7 of light cycle. Dark testing period: hours 4–7 of dark cycle. Percent change from air breathing is shown for: (A) hypoxia (10% O_2_, balanced N_2_) in young mice (~4 months; *n* ****=** **6), (B) hypoxia in middle‐aged mice (~13 months, *n* ****=** **10), (C) hypercapnia (20.93% O_2_, 5% CO_2_, and balanced N_2_) in young mice, (D) hypercapnia in middle‐aged mice, (E) hypoxic hypercapnia (10% O_2_, 5% CO_2_, balanced N_2_) in young mice, and (F) hypoxic hypercapnia in middle‐aged mice. Analysis was performed using a mixed model ANOVA. *Significant main effect of baseline segment for all exposures (*P* < 0.001). Significant main effect of circadian cycle during hypoxia (*P* = 0.019). Mice were excluded if baseline values were > 3SD from the mean (one mouse excluded in each age group). All data are presented as mean ± SD.

Exposure to hypoxia, hypercapnia and hypoxic hypercapnia responses were calculated as a percent change from each of the baseline segments to the average 10‐min response for each gas exposure. There was a main effect of baseline segment for breathing frequency, where a significantly blunted frequency response resulted from using the 10‐min baseline compared to the 30‐sec quiet baseline, for hypoxia, hypercapnia and hypoxic hypercapnia (*P* < 0.001, Fig. 3). Use of the 10‐min baseline for breathing frequency resulted in negative or only slightly positive percent change, whereas greater percent changes for breathing frequency were observed with a 30‐sec baseline. Circadian cycle period also produced a significant main effect during hypoxia (*P* = 0.019) for breathing frequency, where the dark cycle had larger increases in percent breathing rate. There were no differences in percent change for breathing frequency due to age (*P* > 0.05).

**Figure 4:**
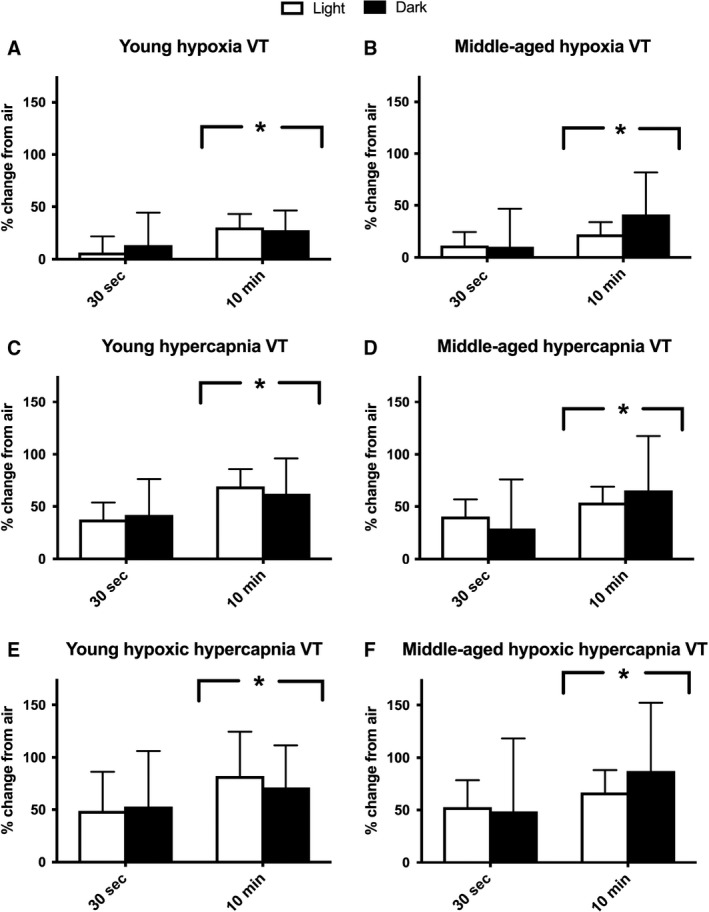
Percent change from air breathing tidal volume (VT) to VT during various gas exposures is larger with the use of a 10‐min baseline. Air breathing was calculated as the 30‐sec of quiet breathing or the 10‐min average at the end of the first hour of air breathing. Each mouse's individual percent (%) change for a given gas exposure (10‐min average) was calculated. Both cohorts were tested in light and dark conditions. Light testing period: hours 4–7 of light cycle. Dark testing period: hours 4–7 of dark cycle. Percent change from air breathing is shown for: (A) hypoxia (10% O_2_, balanced N_2_) in young mice (~4 months; *n* ****=** **7), (B) hypoxia in middle‐aged mice (~13 months, *n* ****=** **11), (C) hypercapnia (20.93% O_2_, 5% CO_2_, and balanced N_2_) in young mice, (D) hypercapnia in middle‐aged mice, (E) hypoxic hypercapnia (10% O_2_, 5% CO_2_, balanced N_2_): (E) in young mice, and (F) hypoxic hypercapnia in middle‐aged mice. Analysis was performed using a mixed model ANOVA. *Significant main effect of baseline segment for all exposures (*P* > 0.05). Mice were excluded if the values were > 3SD from the mean (one mouse excluded in each age group). All data are presented as mean ± SD.

**Figure 5:**
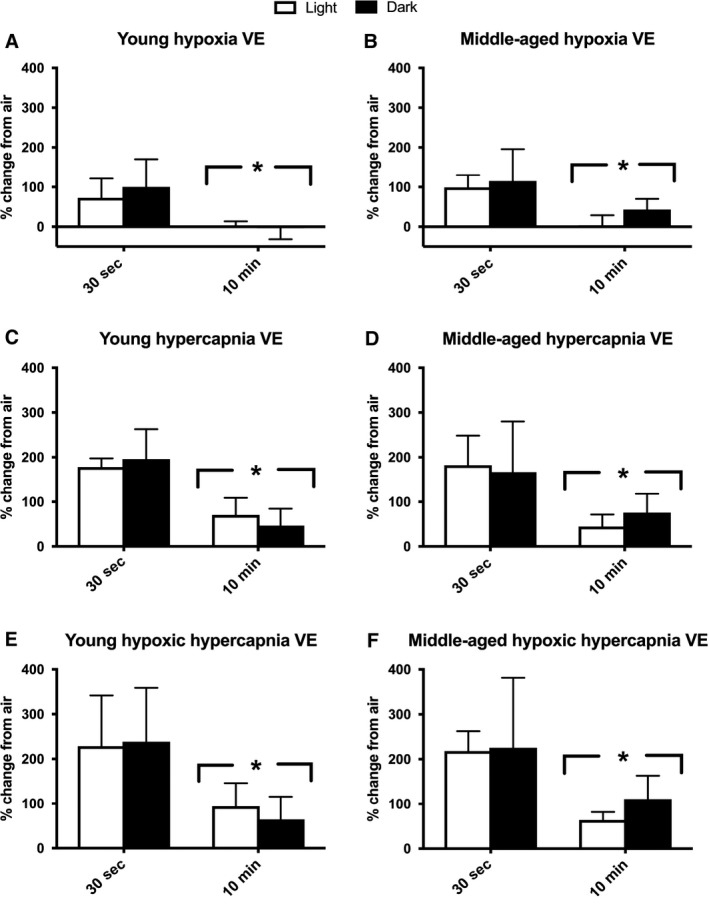
Percent change from air breathing minute ventilation (VE) to VE during various gas exposures is larger with the use of a 30‐sec baseline. Air breathing was calculated as the 30‐sec of quiet breathing or the 10‐min average at the end of the first hour of air breathing. Each mouse's individual percent (%) change for a given gas exposure (10‐min average) was calculated. Both cohorts were tested in light and dark conditions. Light testing period: hours 4–7 of light cycle. Dark testing period: hours 4–7 of dark cycle. Percent change from air breathing is shown for: (A) hypoxia (10% O_2_, balanced N_2_) in young mice (~4 months; *n* ****=** **7), (B) hypoxia in middle‐aged mice (~13 months, *n* ****=** **11), (C) hypercapnia (20.93% O_2_, 5% CO_2_, and balanced N_2_) in young mice, (D) hypercapnia in middle‐aged mice, (E) hypoxic hypercapnia (10% O_2_, 5% CO_2_, balanced N_2_) in young mice, and (F) hypoxic hypercapnia in middle‐aged mice. Analysis was performed using a mixed model ANOVA. *Significant main effect of baseline segment for all exposures (*P* < 0.001). Mice were excluded if values were > 3SD from the mean (one mouse excluded in each age group). All data are presented as mean ± SD.

A similar main effect of baseline segment was detected for VE, as use of a 10‐min baseline resulted in attenuated percent changes when compared to a 30‐sec baseline for each gas exposure (*P* < 0.001; Fig. 4). No main effects were observed for age or circadian cycle segment for VE or VT at baseline. Interestingly, VT had an opposite response for main effect of baseline period used, where the 10‐min baseline resulted in a larger percent change for hypoxia, hypercapnia, and hypoxic hypercapnia (Fig. 5, *P* < 0.05) compared to the 30‐sec baseline. Hence, the larger VT values reported for 30‐sec of quiet breathing baseline blunted the percent change observed for VT in response to these gas exposures.

### VE/VO_2_

VE/VO_2_ during baseline was analyzed in a subset of mice (middle‐aged dark cycle trials) to account for possible differences in metabolic rate (Table 2) between baselines. At baseline, there was no difference in VE/VO_2_ between time segments (*P* = 0.66). Therefore, although the pattern of breathing was different between baseline segments, it is unlikely that arterial blood gases were altered. For hypoxia, we analyzed a 30‐sec time period at the fifth minute as well as a longer segment from minutes 4–10 of gas exposure. VE/VO_2_ was significantly higher during the hypoxic 30‐sec bout (*P* = 0.03). This is not overly surprising given that VE was most pronounced within the fifth minute when the 30‐sec segment was analyzed, compared to the longer time frame which encompassed a slight drop in VE from the maximum values towards the end of the hypoxic bout. We also compared the pattern of breathing during these same time segments of hypoxia to determine if frequency, VT and VE were affected by the time segments analyzed. Frequency, VT and VE were all similar (*P* > 0.05) between 30‐sec (at the fifth minute) and minutes 4–10 of hypoxia (Table 3), although comparing 30‐sec to the entire 10 min of hypoxia did result in higher values at 30‐sec. This finding is due to the “ramping up” of breathing during the first few minutes of hypoxia.

**Table 2:** Comparison of VE/VO_2_ during baseline and hypoxia

Time segment	VE/VO_2_
30 sec: air breathing	183.68 ± 82.31
10 min: air breathing	170.39 ± 39.83
30 sec: hypoxia	400.17 ± 213.27
min 4–min 10: hypoxia	292.86 ± 138.79*

Average VE/VO_2_ of the middle‐aged dark cycle cohort. Baseline values were analyzed during 30‐second (30 sec) and ten‐minute (10 min) time segments for air breathing (20.93% O_2_, balanced N_2_). Hypoxia (10% O_2_, balanced N_2_) VE/VO_2_ was calculated from 30‐sec during the fifth minute of exposure (30 sec) and from minutes 4–10 of exposure (10 min). Measures were analyzed during the dark period from the middle‐aged cohort of mice (~13 months old; *n* ****=** **7). *Significantly different from 30 sec (*P* = 0.038). All data are presented as mean ± SD.

**Table 3:** Comparison of 30‐sec and minute 4–10 pattern of breathing during hypoxia

	Frequency (breath/min)	Tidal volume (mL/breath)	Minute ventilation (mL/min)
30 sec: hypoxia	293 ± 39	0.69 ± 0.28	203.7 ± 81.9
min 4–min 10: hypoxia	289 ± 34	0.69 ± 0.30	200.3 ± 87.2

Average frequency, tidal volume, and minute ventilation during 30‐sec and minutes 4–10 segments of hypoxia. Measures were analyzed during the dark period from the middle‐aged cohort of mice (~13 months old; *n* ****=** **7). For hypoxia, 30‐sec data were analyzed during the fifth minute of hypoxic gas exposure and during minutes 4–10 of exposure. No significant differences between time segments for any measures of breathing were detected (*P* > 0.05). All data are presented as mean ± SD.

## Discussion

Our primary goal was to investigate the possible differences in the use of a tailored, quiet breathing segment compared to a pre‐set time segment for baseline in mice. Additionally, we aimed to determine the use of each baseline for breathing pattern changes of mice exposed to various gas challenges. Overall, these data show that the baseline pattern of breathing is significantly different based on the segment defined as baseline. Circadian cycle also showed an effect on the pattern of breathing, where the dark cycle testing resulted in higher baseline VT measures and higher percent change breathing frequency during hypoxia. However, VE/VO_2_ was similar between air breathing segments in the cohort tested, suggesting that blood gases are maintained in either segment but the pattern of breathing is markedly different. Although not unexpected, baseline segments had a significant effect on the calculated percent change during hypoxic, hypercapnic and hypoxic hypercapnic gas exposure. Hence, we conclude that the protocol used for baseline measures could be enhanced by offering information about mouse behavior and detailing specifics about the segment.

While mouse behavior varied across baselines, we added a proof of concept analysis in one cohort of mice to quantify VE/VO_2_. In the middle‐aged dark group, VE/VO_2_ data were quantified during both baselines to uncover possible ventilation and/or metabolic differences. We observed that although the pattern of breathing is different during 30‐sec and 10‐min of baseline, normalizing to metabolism results in similar VE/VO_2_ values. This finding is similar to typical responses in healthy, control animals that include maintenance of ventilation during various activities. These data also emphasize that mice have many respiratory behaviors that may be exhibited during air breathing. Specific details about behaviors during air breathing, and/or the type of baseline collected are important for data interpretation.

We aimed to document a point by point comparison between baseline breathing and gas exposures. Since breathing during hypoxic exposure and other gas challenges is typically void of additional locomotor movement and sniffing (Fig. 1), the 30‐sec quiet baseline may be more appropriate for comparing to exposures resulting in increased respiratory output. In the middle‐aged dark cohort, VE/VO_2_ was analyzed during hypoxia for 30‐sec and a longer time segment of minute four to minute ten of hypoxia. In this case, VE/VO_2_ was different depending on the time segment analyzed even though frequency, VT and VE were similar across segments. The similar frequency, VT and VE during 30‐sec and a longer segment of hypoxia are in line with our original hypothesis that mouse behaviors are relatively consistent once the new gas has equilibrated in the chamber. It is possible that a 30‐sec baseline versus 30‐sec hypoxic exposure may be the better comparison to ensure that future studies are comparing “apples to apples”. Regardless, the findings suggest that baseline pattern of breathing could impact the magnitude of response to gas challenges; thus, the metrics used for defining baseline should be reported in the literature accordingly. The discrepancies in VE/VO_2_ during 30‐sec and minute 4–10 of hypoxia are likely related to the classic respiratory response during hypoxic gas exposure. Acute hypoxia can lead to an initial increase in VE/VO_2_ followed by a hypoxic depression, similar to the response we observed (Bisgard and Neubauer 1995; Powell et al. 1998). Respiratory depression during longer bouts of hypoxia exposures may be another reason to compare a 30‐sec hypoxic response during the midpoint of hypoxia to a 30‐sec calm baseline with no sniffing and grooming.

With many experimental designs implementing barometric plethysmography in mice, it is critical to document sources of variation between studies so that appropriate comparisons can be drawn. Discrepancies between studies examining mouse pattern of breathing can include those resulting from strain differences (Tankersley et al. 1997; Gonsenhauser et al. 2004) to the type of methodology implemented (Ivy and Scott 2017). Baseline segments can also vary between laboratories and experimental designs, and may play a part in the breathing patterns reported (DeRuisseau et al. 2009; Receno et al. 2018a). VT and/or VE are also altered by factors such as humidity, barometric pressure and temperature (Mortola and Frappell 1998), which can differ between laboratory setups, and could change on a day to day basis, although the Drorbaugh and Fenn equation does take into account the influence of these parameters. One factor that can be streamlined between experimental designs is the use of a similar baseline protocol. Our lab and others have previously made use of quiet breathing baselines, where animals are observed to be awake but with limited/no grooming or sniffing periods (Hickner et al. 2014; Komnenov et al. 2016; Receno et al. 2018a,b). Alternative experimental designs have used baselines where measures are analyzed after a predetermined length of time (DeRuisseau et al. 2009). In the latter scenario, it is likely that more variation will exist, as behavior and activity levels between mice may differ during the baseline segment. This occurrence was evident during baseline testing, as the 10‐min segment showed a larger variation (Table 1) for breathing frequency, VT and VE in most of the animals tested, and had visibly fewer breaths accepted by the Ponemah software (breathing traces shown in Fig. 1A and B). The larger standard deviation is an important consideration due to its impact on detecting statistical differences between and within groups, not only at baseline but for implementation of gas exposures. We reason that comparing a calm baseline to gas exposures is a direct comparison due to the regularity of breathing and mouse behavior across the interventions (Fig. 1). In this case we may be detecting the lowest VE in these mice which should be considered during interpretation. For instance, mice in their home cages tend to go through periods of resting calmly interspersed with more active segments (eating, playing, grooming). We have quantified the calm sections (30‐sec) in the chamber, as the active segments are more challenging to compare across mice in a unique environment for the same time period. As locomotor activity of mice declines with aging (Basso et al. 2016), the use of a calm baseline where mice are stationary would more adequately represent normal cage behavior when undergoing aging plethysmography studies. Experiments using mouse strains with more anxious activities may want to make use of a shorter quiet baseline period, as anxiety may be heightened during testing and result in only short bouts of calm, steady breathing. In these cases, it may be most representative to report both calm and active segments of breathing to encompass multiple behaviors, although we emphasize that calm breathing may be a more direct comparison for responses during gas challenges.

As we report here, initial baseline will determine the magnitude of responses to hypoxia, hypercapnia and hypoxic hypercapnia. Use of the 10‐min predetermined baseline compared to a 30‐sec quiet breathing segment resulted in smaller percent changes to gas exposures for frequency and VE, but larger percent changes in VT. Both frequency and VE were significantly higher during the 10‐min baseline segment when compared to the 30‐sec quiet baseline, which resulted in the blunted percent change response to gas exposures. Interestingly, baseline VT was lower for the 10‐min segment, and ultimately resulted in a larger percent change when compared to 30‐sec of baseline. The lower VT baseline values at 10 min are most likely a compensation for the higher frequency observed in the 10‐min baseline. In this case, the higher frequency could also be a result of sniffing, which would have a lower VT. The lower VT values are in line with another report using a similar baseline (DeRuisseau et al. 2009). Ultimately, the findings surrounding frequency are especially compelling. Frequency may be compared between and across experimental setups since it is not altered by environmental factors such as humidity and temperature. VE and VT calculations require humidity, ambient temperature, body temperature and barometric pressure values to be collected throughout the experiment (Mortola and Frappell 1998).

While physiological circadian differences have been reported in many systems (Aschoff and Pohl 1970; Refinetti and Menaker 1992; Rodrigo and Denniff 2016), including pattern of breathing (Seifert and Mortola 2002; Ishiguro et al. 2006; Ohshima et al. 2007; Quindry et al. 2016), there is increased interest in this phenomenon. More studies are being initiated on the topic of circadian cycle differences in physiology. Particular emphasis about the behavior of conscious mice during these types of investigations is vital to data interpretation. Since mice are nocturnal, the dark period can be heavily made up of active behaviors (grooming, exploring, etc.) and these details are essential information for breathing studies. In our experience, we found that obtaining a quiet baseline was more challenging during the dark cycle, especially when attempting to get longer (2–5 min) continuous segments for analysis. The time to a 30‐sec baseline was longer in the dark versus light cycle (82 ± 26 min vs. 54 ± 20 min, *P* = 0.036). Anecdotally, mice were more prone to having shorter bouts of restful periods following long periods of activity (e.g., grooming, sniffing, exploring the chamber). A recent study by Teske et al. (2014) reported that spontaneous physical activity was increased in mice during both the dark and light cycles on the first day within a new environment, versus rats who only had increased activity during the light cycle. This publication is a significant finding, as the data suggests that rat exploratory behavior is different from mice in the light and dark cycles. While taking this information into consideration, we are confident that quiet baseline data retrieved from the dark and light cycle of these mice are truly indicative of quiet, steady, awake breathing, and no sniffing/grooming behavior. It is not possible for a mouse to have calm breathing while moving about in the chamber; the increased movement either results in heightened breathing or artifact. Both of these possibilities result in a specific flow tracing that is detected by an experimenter trained in breathing analysis, and can be further aided with the help of detailed behavioral notes taken during data collection. Viewing breathing traces was imperative for obtaining our quiet breathing segments during the dark and light cycles. Importantly, a previous study by Kabir et al. (2010) concluded that lack of locomotor movement alone is not indicative of a calm breathing rat. They reported increases in breathing frequency in rats which had heightened arousal from various stimuli, even though rats were stationary. Hence, observing consistent breathing patterns flanked by periods of activity (Fig. 1) on the Ponemah traces gave the best indications of calm, quiet breathing in an animal that was awake.

We report VT to be significantly greater during the dark period of the circadian cycle during air breathing, with additional differences in frequency and VE depending on the time segment used. The percent change for breathing frequency was also higher in the dark cycle, but only in response to hypoxic challenge, a finding similar to that by Ohshima et al. (2007). Although body temperature can influence VT and VE, we did not observe differences in body temperature across the circadian cycle during unrestrained barometric plethysmography (36.7 ± 0.6 vs. 36.9 ± 0.7°C). We also measured body temperature in the home cage (35.6 ± 0.9 vs. 36.4 ± 1.5°C, light vs. dark, *P* = 0.128); the variation in body temperature across circadian cycle in the home cage is more typical. The discrepancies between body temperatures in the home cage versus chamber are likely due to the habituation to the chamber. For our study, larger patterns of breathing values during the dark cycle show that circadian cycle variation still occurs, even when quiet baseline measures are collected. Moreover, it is imperative to achieve a period of quiet breathing before exposing mice to challenges, as the magnitude of response is masked by use of a more active baseline. Future investigations may also take into account the active behaviors within the dark cycle (e.g., sniffing) and quantify the response (Youngentob 2005) within the unrestrained barometric plethysmography chamber. Ultimately, it is important to acknowledge the type of baseline collected can have implications for reported results and interpretations.

Behavior and anxiety in the mouse model has been shown to vary depending on strain or sex (O'Leary et al. 2013; Tanaka 2015; Shanksy 2018). We investigated baseline in male mice; it is possible that females and/or other strains may be more/less excitable in the chamber, and using a comprehensive baseline could be useful in such studies to differentiate active and calm breathing. A quiet baseline designation would help to ensure that these behavioral differences aren't confounding breathing pattern interpretations, and that measures are truly indicative of the physiological response from quiet breathing versus the heightened (observed) baseline response that may come from being in the chamber. In future studies, both the active and quiet baseline values could be reported to describe these segments in other strains of mice.

## Conclusion

Overall, the use of a quiet baseline is a worthy consideration when utilizing unrestrained barometric plethysmography to characterize breathing patterns of mice. A quiet breathing baseline resulted in larger magnitudes of change for frequency and VE as a result of gas exposures, but an attenuated VT response. Normalizing VE to metabolism showed that while baseline pattern of breathing differs across air breathing segments, blood gases are likely maintained since VE/VO_2_ values are similar. Based on these data, we recommend an unrestrained barometric plethysmography experimental design that accounts for mouse behavior. Importantly, the results stress the need to report specific information pertaining to baseline, regardless of what type is used, to ensure appropriate comparisons between studies.

## Conflict of Interest

The authors have no conflict of interest to disclose.
